# Trends in Maternal Mortality Ratio in a Tertiary Referral Hospital and the Effects of Various Maternity Schemes on It

**Published:** 2015-06

**Authors:** Harpreet Kaur, Sharanjit Kaur, Sukhwinderjit Singh

**Affiliations:** 1 Department of Obstetrics and Gynaecology, Gurugobind Singh Médical Hospital, Faridkot, Punjab, India; 2 Department of Obstetrics and Gynaecology, University College of Nursing, Faridkot, Punjab, India; 3 Department of Orthopedics, Civil Hospital, Kot-kapura, Punjab, India

**Keywords:** Maternal Mortality, Maternal Mortality Ratio, Hemorrhage, Anemia, Sepsis, Eclampsia

## Abstract

**Objective:** To analyze the trend in maternal mortality ratio in a tertiary care centre and the effect of various maternity schemes on it.

**Materials and methods:** Retrospective analysis of all maternal deaths occurring in the Guru Gobind Singh Medical College & Hospital, Faridkot, Punjab, India was done from Jan 2010 to Dec 2012. Every maternal death was scrutinized from various aspects like direct cause of death, age, locality, antenatal care and gestational age.

**Results:** The total number of deliveries has risen from 957 in 2010 to 1063 in 2012 at the same time the maternal mortality ratio has increased from 835.94 in 2010 to 2054.55 per one live birth in 2012. Haemorrhage (24.12%) and sepsis (18.9%) were the most common causes of death followed closely by pregnancy induced hypertension including eclampsia (15.5%). Anemia (12.06%) contributed to the most common indirect cause of death.

**Conclusion:** Implementation of the various maternity schemes has had no significant impact on the profile of dying mothers. There is a need to stress the importance of good antenatal care in reducing Maternal Mortality Ratio.

## Introduction

Maternal mortality is defined as the number of direct and indirect maternal deaths per 1 lac live births up to 42 days after termination of pregnancy. The maternal mortality in India declined from 254 during 2004-2006 to 230 (1). In Punjab the maternal mortality ratio is 172/1,00,000 live births. The government has launched various schemes like Janani Suraksha Yojna, Mata Kaushalaya scheme to promote institutional deliveries.

For improvement in mortality a planned strategy is required by government. The real change will come only with the involvement of public health sector from villages to metros (2).

Institutional maternal mortality rates are 2-10 times higher as compared with field surveys because most of the seriously ill patients are referred to the tertiary care centre (3).

Although more pregnant women are accessing the health services, lack of adequate trained and motivated personnel at these sites probably resulted in the unfortunate women being picked up and referred late to a tertiary centre.

## Materials and methods

Retrospective analysis of all maternal deaths occurring in the Department of Obstetrics & Gynecology of Guru Gobind Singh Medical College & Hospital, Faridkot was done from Jan 2010 to Dec 2012. Every maternal death was scrutinized from various aspects like direct cause of death, age, locality, antenatal care and gestational age.

The study was carried out with the purpose of analyzing the impact of these schemes on quality of antenatal care and maternal mortality.

## Results

As shown in Table 1 the total number of deliveries has risen from 957 in 2010 to 1063 in 2012. The maternal mortality ratio has also simultaneously increased from 835.94 to 2054.55 per/lac live births.

Table 2 shows age, parity and residence profiles of patients

As shown in Table 3, hemorrhage (24.12%) which included atony, APH and uterine rupture and sepsis (18.96%) were the two most common direct cause of death followed closely by pregnancy induced hypertension including eclampsia (15.5%).

DIC was the cause of death in 8.6% of cases and amniotic fluid embolism and pulmonary embolism were the suspected causes in 1.72% and 5.17% of cases respectively.

Anemia (12.06%) contributed to the most common indirect cause of death other indirect causes which contributed to maternal mortality was cardiovascular disease and hepatitis in 5.17% each. Acute renal failure contributed to 3.44% of cases. Only one case of malaria was reported (Table 3).

**Table 1:** Maternal Mortality ratio

Year	Maternal Deaths	Live Births	MM. Ratio per/lac live births
**2010**	**8**	**957**	**835.94**
**2011**	**19**	**803**	**2366.12**
**2012**	**31**	**1063**	**2916.20**
**Total**	**58**	**2823**	**2054.55 per lac**

**Table 2:** Maternal deaths and its characteristics

	Maternal Deaths	Percentage
**Age**		
** 10-20 years **	**Nil**	
** > 20-30 years **	**44**	**75.86**
** > 30-40 years **	**13**	**22.41**
** > 40 years **	**1**	**1.72**
**Parity**		
** Primi**	**16**	**27.58**
** Multi**	**42**	**72.41**
**Antenatal Care **		
** Booked**	**13**	**22.41**
** Un-booked**	**45**	**77.58**
**Locality**		
** Rural**	**38**	**65.51**
** Urban**	**20**	**34.48**
**Gestation (Weeks)**		
** < 20**	**7**	**12.06**
** 20-37**	**18**	**31.03**
** > 37**	**6**	**10.30**
** Post Partum**	**27**	**46.50**

**Table 3:** Causes of Maternal Deaths

Causes	Frequency (No.)	Percentage (%)
**Direct Causes**		
**Haemorrhage**	**14**	**24.12%**
**Atony**	**6**	**10.34%**
**Antepartum Haemorrhage**	**6**	**10.34%**
**Uterine Rupture**	**2**	**3.44%**
**Ectopic**	**0**	**-**
**Following caesarean**	**0**	**-**
**Molar Pregnancy **	**0**	**-**
**DIC**	**5**	**8.6**
**Adherent Placenta**	**0**	**-**
**Sepsis**	**11**	**18.96**
**Pregnancy Induced Hypertension **	**9**	**15.51**
**Amniotic Fluid Embolism**	**1**	**1.72**
**Pulmonary Embolism**	**3**	**5.17**
**Acute Uterine Inversion**	**0**	**-**
**Indirect Causes**		
**Anaemia**	**7**	**12.06**
**Cardiovascular Disease **	**3**	**5.17**
**Hepatitis**	**3**	**5.17**
**Asthma**	**0**	**-**
**Epilepsy**	**0**	**-**
**Acute renal failure**	**2**	**3.44**
**Terminal Stage of Malignancy**	**0**	**-**
**Malaria**	**1**	**1.72**

## Discussion

Being a predominantly referral hospital, most of the women coming have risk factors and many are in a moribund state. As such the maternal mortality ratio is always high in a tertiary care centre as compared to periphery (4).

Unfortunately Janani Suraksha Yojna Scheme and other schemes have put an undue stress on institutional deliveries without making an effort to promote the importance of good antenatal care and reducing maternal mortality and morbidity by early detection and treatment of preventive causes.

Table 2 shows age, parity and residence profiles of patients implying that our poor village girls are still married early and die young, maternity remaining a preventable cause of these tragic deaths (5). Totally 70-80% of deaths were amongst UN booked mothers as shown in Table 2. This was similar to other studies (6).

Poverty and illiteracy, casual acceptance of child bearing together with a shortage of trained and more dedicated health professional remains a major hurdle in providing good antenatal care (4).

The common direct causes of maternal mortality are hemorrhage, sepsis, pregnancy induced hypertension including eclampsia which if not absolutely preventable are well treatable. Anemia which is the most common indirect cause of death is absolutely preventable and it reveals the dismal nature of primary prevention from childhood through adolescence into pregnancy.

## Conclusion

The schemes need to be remodeled so that the incentives may be phased out to cover antenatal care as well. There is also a need to make these incentives limited by the number of living issues, for in practice they promote multiparty and associated risks. Death reviews, attended by all personnel (health and administrative, public and private) involved in the care of pregnant women should be held, accountability discussed and fixed (7).

Early intensive efforts to improve family planning, to control fertility choices, to provide safe abortion and integrated maternal health services are the most important interventions to reduce pregnancy related mortality (8).
